# Three-dimensional bicontinuous nanoporous materials by vapor phase dealloying

**DOI:** 10.1038/s41467-017-02167-y

**Published:** 2018-01-18

**Authors:** Zhen Lu, Cheng Li, Jiuhui Han, Fan Zhang, Pan Liu, Hao Wang, Zhili Wang, Chun Cheng, Linghan Chen, Akihiko Hirata, Takeshi Fujita, Jonah Erlebacher, Mingwei Chen

**Affiliations:** 1 Advanced Institute for Materials Research, Tohoku University, Sendai, 980-8577 Japan; 2 Mathematics for Advanced Materials-OIL, AIST-Tohoku University, Sendai, 980-8577 Japan; 3 State Key Laboratory of Metal Matrix Composites, School of Materials Science and Engineering, Shanghai Jiao Tong University, 200030 Shanghai, China; 4 Department of Materials Science and Engineering, The Johns Hopkins University, Baltimore, MD 21218 USA

## Abstract

Three-dimensional bicontinuous open (3DBO) nanoporosity has been recognized as an important nanoarchitecture for catalysis, sensing, and energy storage. Dealloying, i.e., selectively removing a component from an alloy, is an efficient way to fabricate nanoporous materials. However, current electrochemical and liquid-metal dealloying methods can only be applied to a limited number of alloys and usually require an etching process with chemical waste. Here, we report a green and universal approach, vapor-phase dealloying, to fabricate nanoporous materials by utilizing the vapor pressure difference between constituent elements in an alloy to selectively remove a component with a high partial vapor pressure for 3DBO nanoporosity. We demonstrate that extensive elements, regardless of chemical activity, can be fabricated as nanoporous materials with tunable pore sizes. Importantly, the evaporated components can be fully recovered. This environmentally friendly dealloying method paves a way to fabricate 3DBO nanoporous materials for a wide range of structural and functional applications.

## Abstract (web summary)

Conventional dealloying methods to produce bicontinuous open nanoporous structures for catalysis are limited to very few alloys and produce chemical waste. Here, the authors develop a green process, vapor-phase dealloying, to selectively remove high partial vapor pressure components and create nanoporosity in a wide range of alloys.

## Introduction

Dealloyed nanoporous materials have attracted great attention because of their unique three-dimensional bicontinuous open (3DBO) porous structure with a large specific surface area and high electrical/thermal conductivity for applications in catalysis^1–5^, surface-enhanced Raman scattering^6–8^, actuation^9^, and energy storage and conversion^10–13^. The most common method to fabricate nanoporous metals is electrochemical dealloying by which a less-noble component is selectively dissolved from a precursor alloy by virtue of the standard electrode potential difference between constituent elements^14–18^. A number of noble and easily-passivated nanoporous metals, such as Pt, Au, Pd, Ag, Cu, and Ni, have been successfully fabricated from the binary alloy precursors^19–24^. However, this method cannot be used to fabricate nanoporous metals with relatively high chemical activity. Recently, a liquid-metal dealloying method has been developed to produce nanoporous Ti, Nb, Ta, Si, etc., through the miscibility difference between alloy components and metallic melts^25–29^. However, chemical etching is still required to remove the residual melts which are solidified in the pore channels. Moreover, the high dealloying temperatures of the liquid-metal dealloying usually lead to the coarsening of porous structures and prevent the formation of nanoporosity. In addition to the fact that only a small number of elements can be fabricated into a porous structure by either electrochemical or liquid-metal dealloying, both of these methods have two common downsides for practical applications: producing chemical waste during etching and difficult recovery of dissolved metal components from electrolytes and liquid-metal solvents, which may cause severe environmental issues and thus are not economical and sustainable^30^.

In this study, we developed a vapor-phase dealloying (VPD) method by utilizing the vapor pressure difference between solid elements to selectively evaporate a component from an alloy. This method can be applied to a variety of elements from less-noble metals to inorganic elements regardless of their chemical, and electrochemical activity and electric conductivity. The elements with low melting point can also be fabricated using a low dealloying temperature by reducing dealloying pressures. Importantly, the sublimated elements can be fully recovered and do not cause any environmental issues. Thus, VPD is a universal, cost-saving, and environmentally friendly method for fabricating 3DBO nanoporous materials.

## Results

### Precursor alloy fabrication and VPD method

**Fig. 1:**
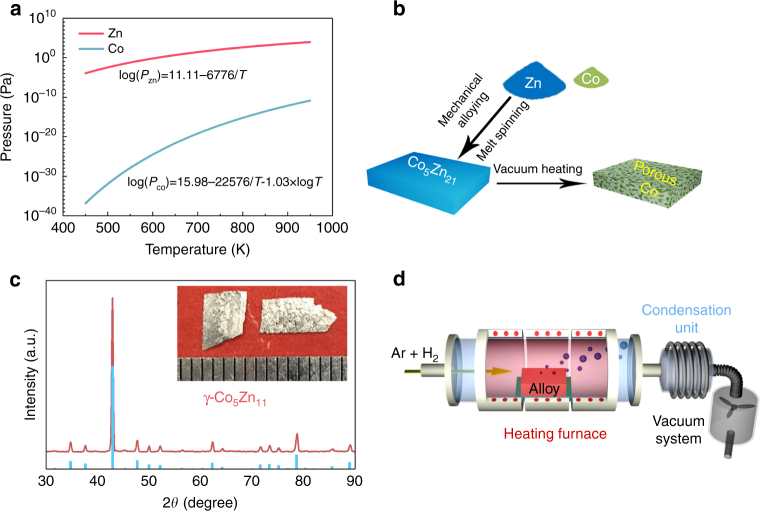
Vapor-phase dealloying and preparation of a prototype Co_5_Zn_21_ precursor. **a** The relation between temperature and saturated vapor pressure of zinc and cobalt in a prototype Zn–Co alloy system. **b** The schematic illustration of the fabrication process of nanoporous cobalt. **c** XRD pattern of Co_5_Zn_21_ precursor ribbons and corresponding PDF standard card of γ-Co_5_Zn_21_. The inset shows a photo of Co_5_Zn_21_ precursor ribbons. **d** The schematic of the high-vacuum recyclable vapor-phase dealloying system

A Co_5_Zn_21_ (atomic ratio) alloy is used as a model system to demonstrate the VPD method. Figure 1a shows the temperature dependence of the saturated vapor pressures of zinc and cobalt^31^. There is a large difference in the saturated vapor pressures between zinc and cobalt in a wide temperature range, indicating that zinc can be selectively removed from the Co_5_Zn_21_ alloy by optimizing the processing temperature and vacuum, whereas Co is stable and does not sublimate. The precursor Co_5_Zn_21_ alloy was prepared by mechanically milling Co (99+%, Nilaco Co.) and Zn (99.85%, Nilaco Co.) powders at room temperature (Fig. 1b). These uniformly mixed powders were further melted under Ar protection for the fabrication of thin Co_5_Zn_21_ ribbons by melt spinning (see inset of Fig. 1c). Energy-dispersive spectroscopy (EDS) mappings show that cobalt and zinc are homogeneously dispersed in the Co_5_Zn_21_ alloy (Supplementary Fig. 1). X-ray diffraction (XRD) of the Co_5_Zn_21_ ribbon reveals the single cubic phase of γ-Co_5_Zn_21_, as shown in Fig. 1c. Electron backscatter diffraction (EBSD) inverse pole figure mapping reveals that the grain size of the precursor alloy is about 10 μm (Supplementary Fig. 2). Figure 1d schematically illustrates the home-built vacuum dealloying system. It is comprised of a high-temperature tube furnace with a maximum heating temperature up to 1573 K, a condensation unit to recover the evaporated zinc and a vacuum system to keep the working chamber at adjustable vacuum conditions (100–6 × 10^−3^ Pa). In particular, the condensation unit allows the recycling of evaporated materials within the system (Supplementary Fig. 3).

### Fabrication of nanoporous cobalt

**Fig. 2:**
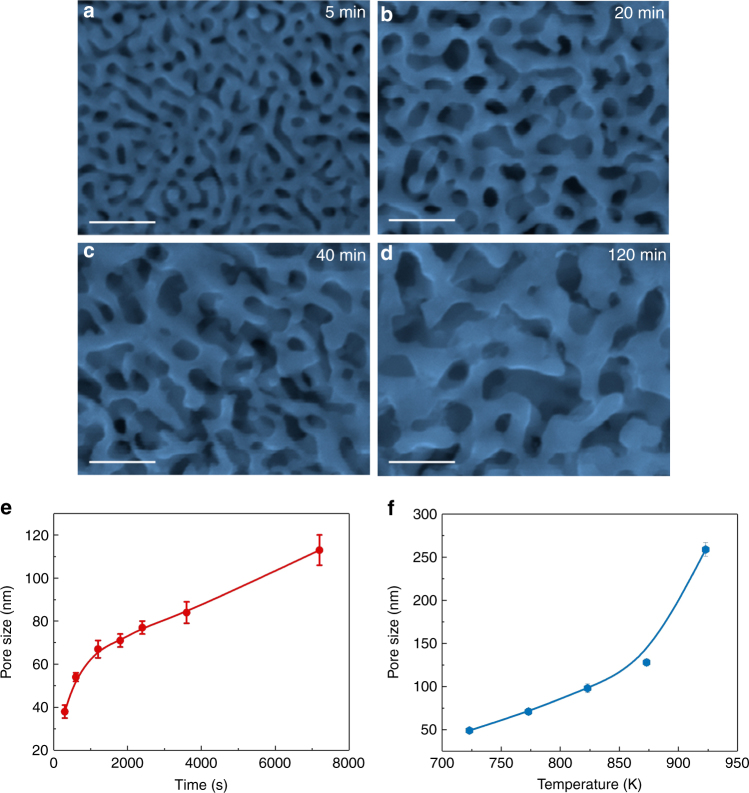
Microstructure characterization of nanoporous cobalt under low-vacuum. **a**–**d** SEM images of nanoporous cobalt with various dealloying time from 5 to 120 min at 773 K and 100 Pa. **a**–**d** Scale bars, 200 nm. **e** The evolution of pore size with dealloying time. **f** The relation between pore size and dealloying temperature at a fixed dealloying time of 30 min

Figure 2a–d show the formation and evolution of nanoporous cobalt with a series of dealloying periods from 5 to 120 min at a constant temperature of 773 K and pressure of 100 Pa. The cross sections of the dealloyed samples are presented in Supplementary Fig. 4. A bicontinuous porous structure can be seen on the sample surface after dealloying for 5 min (Fig. 2a) and the average zinc concentration decreases to 25.3 at.%. The cross-sectional SEM image shows that dealloying only occurs on the surface region of the precursor ribbon to a depth of 5 μm in the short dealloying period (Supplementary Fig. 4a). After dealloying for 20 min, the porous structure has evolved to the entire sample thickness (Fig. 2b; Supplementary Fig. 4b) and the concentration of residual zinc is only about 6 at.% (Supplementary Fig. 5). With further increasing dealloying time, more zinc atoms escape from the nanoporous structure, and both pore and ligament sizes gradually coarsen as shown in Fig. 2a–d. The average pore size, measured by a rotationally averaged fast Fourier transform method^32^, increases from about 40 to 110 nm with a dealloying time from 5 to 120 min (Fig. 2e). The evolution of nanoporosity also strongly depends on dealloying temperatures. At a constant dealloying time of 30 min, the pore and ligament sizes increase from 50 to 250 nm with temperature increasing from 723 to 923 K (Fig. 2f; Supplementary Fig. 6). The temperature and time dependence indicates that, in principle, the kinetics of the VPD is controlled by the competition between the evaporation rate of Zn, which increases quickly with temperature increasing and environmental pressure decreasing, and a surface diffusion of remained Co, similar to electrochemical dealloying^33–35^.

### High-vacuum dealloying process

**Fig. 3:**
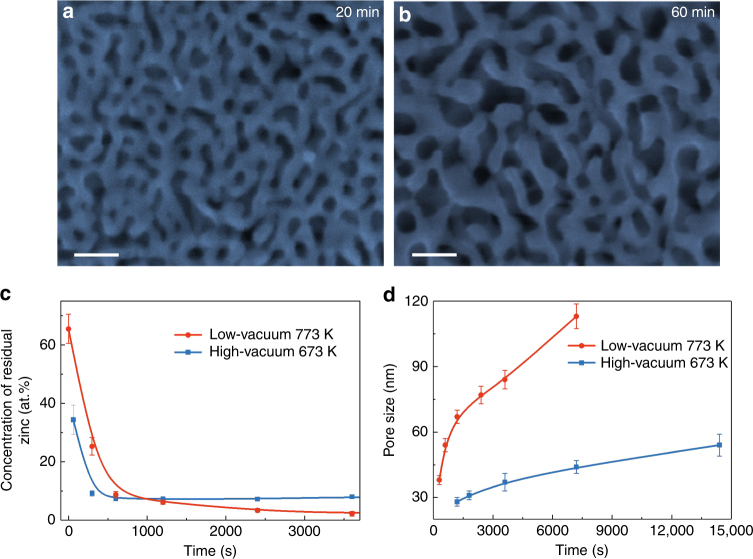
Microstructural characterization of nanoporous cobalt under high-vacuum. **a**, **b** SEM images of nanoporous cobalt with dealloying time of 20 and 60 min at 673 K and 6 × 10^−3^ Pa. **a**, **b** Scale bars, 100 nm. **c** The relation between the concentration of residual zinc and dealloying time for both low- and high-vacuum dealloying conditions. **d** Correlation between pore size and dealloying time for different dealloying conditions

In addition to temperature and time, the environmental pressures at which dealloying is conducted also play an important role in nanopore formation and have a significant influence on dealloying kinetics. Owing to the large equilibrium vapor pressure difference between pure Zn and Co, it can be qualitatively estimated that Zn has a much higher partial vapor pressure than that of Co in the Zn-rich alloy according to Raoult’s law. A higher vacuum (i.e., a lower environmental pressure) is expected to increase the vaporization rate of Zn and allow the fabrication of nanoporous structures at a relatively lower temperature and shorter time, which benefits the formation of ultrafine nanoporous structures by limiting the surface diffusion of Co. Figure 3a shows the SEM image of nanoporous cobalt prepared by dealloying at 673 K and 6 × 10^−3^ Pa for 20 min. A nanoporous structure with a small pore size of about 28 nm is obtained, falling into the pore size range of electrochemically dealloyed nanoporous metals. Chemical analysis suggests that only about 7 at.% zinc is left in the nanoporous cobalt (Fig. 3c). Further increasing dealloying time to 60 min, retaining the high vacuum, the pore size slightly increases to about 36 nm (Fig. 3b) with nearly no change in the concentration of residual zinc. In contrast to the high-vacuum dealloying, a porous structure cannot be produced at the pressure of 100 Pa and 673 K (Supplementary Fig. 7). Therefore, high vacuum significantly promotes the dealloying kinetics of VPD and the formation of an ultrafine nanoporous structure.

We plotted the time dependence of residual zinc concentration at 100 Pa and 773 K as well as at 6 × 10^−3^ Pa and 673 K in Fig. 3c. The high-vacuum dealloying condition exhibits a much faster volatilization rate during the first 600 s even though the temperature is 100 K lower. After 1000 s, the concentration of the zinc at 6 × 10^−3^ Pa remains nearly constant whereas it keeps slowly decreasing at 100 Pa and 773 K. It appears that the enhanced dealloying kinetics primarily originates from a higher volatilization rate of zinc in higher vacuum when the concentration of zinc is higher in the precursor alloy at the early stage of dealloying. The fact that high temperatures benefit the continuous volatilization of low concentration zinc indicates the diffusion of Co is important in the late stage of dealloying, which corresponds to the coarsening of nanopores and ligaments. Figure 3d presents the relationship between the average pore size and the dealloying time at the two different dealloying conditions. The growth rate of nanoporous cobalt in high-vacuum condition is much lower than that in low-vacuum condition due to the lower dealloying temperature.

## Discussion

**Fig. 4:**
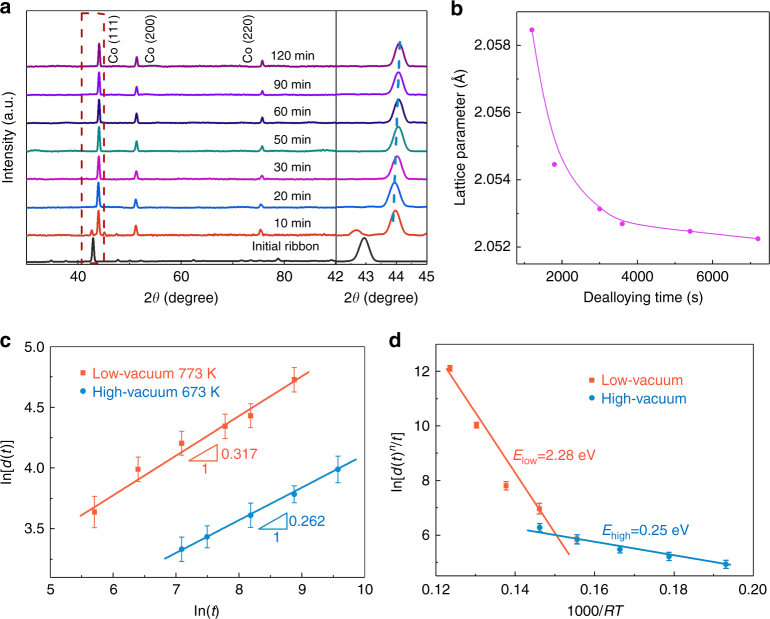
Structure and physical properties of nanoporous cobalt. **a** XRD patterns of nanoporous cobalt prepared with various dealloying time at 773 K and 100 Pa. **b** Evolution of lattice parameter of nanoporous Co with different dealloying time at 773 K and 100 Pa. **c** Measurements of the coarsening exponents from the plots of ln[*d*(*t*)] vs ln(*t*) for low- and high-vacuum dealloying conditions. **d** The estimated activation energies for the nanoporous cobalt formation and coarsening at different dealloying conditions

Figure 4a shows XRD patterns of the samples dealloyed at 100 Pa and 773 K at times from 0 to 120 min. The diffraction peaks of the precursor Co_5_Zn_21_ phase at 2*θ = *42.934° and 78.82° gradually decrease with increasing dealloying time and finally disappear after 20 min. Instead, the characteristic peaks of the face-center-cubic cobalt appear and progressively shift to the high angles. The lattice parameter of the resulting nanoporous cobalt at various dealloying time is calculated from the (111) peak by Bragg’s law as shown in Fig. 4b. It reduces by about 0.3% after complete dealloying for 120 min, indicating the gradual loss of residual Zn from the nanoporous Co even after nanopore formation. We noticed that significant volume shrinkage takes place during the VPD, similar to electrochemical and liquid-metal dealloying. The cross-section thickness is reduced by about 55%, from 60 μm of Co_5_Zn_21_ to 27 μm of nanoporous cobalt after dealloyed at 773 K for 30 min (Supplementary Fig. 8). The shrinkage appears to be isotropic along three dimensions (Supplementary Fig. 9). The volume shrinkage results from the loss of Zn and the relaxation of remained Co. As a higher vacuum (a lower environmental pressure) promotes Zn volatilization and a lower dealloying temperature restricts Co diffusion, the dealloying at higher vacuum and lower temperatures can effectively prevent volume shrinkage and allow the fabrication of nanoporous Co with a larger porosity, lower density and smaller pore sizes (Supplementary Fig. 10).

The coarsening rates of nanopores and cobalt ligaments are similar and can be well described by the following equation^33, 36^:

1 $${d}\left( t \right)^n = k_0t\exp \left( {\frac{{ - E}}{{RT}}} \right) = KtD_{\rm s},$$

where the diffusivity $D_{{\rm s}} = D_0\exp \left( {\frac{{ - E}}{{RT}}} \right)$; *d*(*t*) is the pore size at dealloying time *t*; *k*_0_, *K*, and *D*_0_ are constants and *k*_0_ = *KD*_0_; *n* is the coarsening exponent; *R* is the gas constant; *T* is the dealloying temperature; and *E* is the activation energy for nanopore formation and coarsening. A good linear relationship between ln[*d*(*t*)] and ln(*t*) can be obtained for both high vacuum (6 × 10^−3^ Pa) and low-vacuum (100 Pa) dealloying conditions (Fig. 4c), verifying that the growth of nanoporous cobalt during VPD is a thermal activation-controlled process. The scaling exponent *n* for the low-vacuum condition is determined to be 3.15. This value is close to *n* = 3, indicating that bulk diffusion governs the dealloying process^35^. In contrast, for high-vacuum condition, we find *n* = 3.82. It is close to the scaling exponent *n* = 4, suggesting that the surface diffusion dominates the dealloying process^33, 35^.

On the basis of the linear fit between ln[*d*(*t*)^*n*^/*t*] and (*RT*)^−1^, the activation energy for the formation and coarsening of nanoporous cobalt at high (6 × 10^−3^ Pa) and low-vacuum (100 Pa) conditions are measured to be ~0.25 and ~2.28 eV, respectively (Fig. 4d). These values are close to the activation energies of the surface diffusion of Co (0.14 eV) and bulk diffusions of Zn in Co (2.76 eV), respectively^37, 38^, which is consistent with the analysis of the coarsening exponent and further demonstrates that the dealloying is mainly controlled by bulk diffusion at low vacuum of 100 Pa and by surface diffusion at the high vacuum of 6 × 10^−3^ Pa. The pressure dependent micro-mechanisms indicate that the interplay between Zn volatilization and Co diffusion plays a key role in nanopore formation and coarsening. At high vacuum, the evaporation of Zn is fast enough to leave behind under-coordinated Co atoms on the vacuum/sample interfaces and the formation and coarsening of Co ligaments is dominated by the surface diffusion of Co atoms with a lower activation energy. At lower vacuum and higher environmental pressure, the surface diffusion process is much faster than building a sufficiently high partial vapor pressure of Zn at the dealloying frontier for selectively evaporating Zn. The dealloying process is thus controlled by Zn volatilization which is limited by the slow bulk diffusion of Zn in Co.

It is known that the surface oxidation can dramatically reduce the kinetics of nanopore formation and coarsening as demonstrated by oxide coated nanoporous gold^39^ and passivation of nanoporous Ni^40^. To verify the possible influence of surface oxidation on the dealloying kinetics of nanoporous Co, we employed a high-resolution scanning transmission electron microscope equipped with an electron energy-loss spectrometer (EELS) to investigate the surface structure and chemistry of Co ligaments. No detectable oxide layers of oxygen segregation can be seen from the EELS chemical mappings of nanoporous cobalt samples prepared at high and low vacuum (Supplementary Figs. 11 and 12). This is consistent with the facts that VPD takes place in a reduction atmosphere at high temperatures because Zn vapor itself is a strong reducing medium, in addition to the usage of protection gas (pure Ar or Ar + H_2_). It is worth noting that the VPD process is not caused by any gas–gas or gas–solid chemical reactions even with the presence of a very small amount of hydrogen in the protection flowing gases, which is fundamentally different from the newly developed drying dealloying method, which selectively removes one component from AgAu alloys by utilizing gas oxidation^21^.

Because VPD simply utilizes the vapor pressure difference to selectively remove one component from an alloy, we have a broad range of opportunities to make a precursor alloy suitable for dealloying by pairing two elements with different saturated vapor pressures. By utilizing this method, we have successfully fabricated nanoporous Si, Ti, and Ni (Supplementary Fig. 13), in addition to Co. In principle, all stable solid elements in the periodic table can be fabricated as a nanoporous material by VPD through precursor design and dealloying parameter optimization.

In summary, we report a universal dealloying method to fabricate 3DBO nanoporous materials. By using Zn–Co as a prototype system, we demonstrated that 3DBO nanoporosity of Co can be achieved by VPD and the pore size can be tailored to a wide size range from tens of nanometers to micrometers by controlling dealloying temperature, time and pressure. In particular, the dealloying pressure significantly changes the rate-limiting dealloying mechanisms from a low-vacuum bulk diffusion controlled process to a high-vacuum surface diffusion controlled process. Because the VPD does not involve any chemical or electrochemical processes, the resulting nanoporous materials can be fabricated from a broad range of elements regardless of their chemical stability and electric conductivity. Importantly, the evaporated components can be fully recovered in the vacuum dealloying system and thus the method is free of chemical pollution. This environmentally friendly and highly efficient dealloying method paves a new way to fabricate and design bicontinuous nanoporous materials for a wide range of structural and functional applications.

## Methods

### Sample preparation

The binary Co–Zn alloy with a nominal composition of Co_5_Zn_21_ (atomic%) was prepared by ball milling (Planetary Mono Mill Pulverisette 6 classic line, Fritsch) with a uniform mixture of Co (99+%, Nilaco Co.) and Zn (99.85%, Nilaco Co.) powders under the protection of Argon at room temperature. Co_5_Zn_21_ ribbons were fabricated by melt spinning with a single Cu wheel at the rotational speed of ~1 krpm. For the low-vacuum dealloying, the pressure of chamber was below 1 Pa by running rotary pump over 30 min, and followed by Ar (250 sccm) flowing for 20 min to further remove oxygen before heating process. Then the precursor Co_5_Zn_21_ ribbons were treated at a series of temperatures under a mixed atmosphere of H_2_ (10 sccm) and Ar (250 sccm) with the pressure ~100 Pa. For comparison, pure Ar without H_2_ was also used as the protection gas and no obvious difference in the dealloying kinetics can be seen. For high-vacuum dealloying, an extra turbo-molecular pump was used to maintain the pressure at 6 × 10^−3^ Pa during dealloying. Ewards Pirani 502 is used to measure low vacuum and Granville Phillips UHV 350 Ion Gauge Controller is used to measure high vacuum. Nanoporous Ti, Ni, and Si were prepared by using the precursor alloys with nominal compositions of Ti_2_Zn_8_, Ni_2_Zn_11_, and Si_2_Zn_8_ (atomic%), respectively. The fabrication methods for precursor alloys and dealloying methods for nanoporous structure are the same as Co_5_Zn_21_.

### Characterization

The crystal structures of Co_5_Zn_21_ ribbons and dealloyed nanoporous Co were characterized by X-ray diffraction with Co–Kα radiation (Rigaku SmartLab 3 kW). The microstructure, chemical composition and grain size of the specimens were investigated using field-emission scanning electron microscope (JEOL JIB-4600F, 15 keV) equipped with an X-ray energy-dispersive spectroscopy (EDS) and an electron backscatter diffraction (EBSD) imaging system. The structural and chemical analysis was characterized by a JEOL JEM-2100F TEM/STEM system with double Cs-correctors (operated at 200 kV).

### Data availability

All relevant data are available from the authors on request.

## Electronic supplementary material

Supplementary Information
