# Supplementary material for: Three-dimensional bicontinuous nanoporous materials by vapor phase dealloying
Source: Nat Commun. 2018 Jan 18;9:276. doi: 10.1038/s41467-017-02167-y (PMC5773601; doi:10.1038/s41467-017-02167-y)
Supplement: Supplementary file 1 — Supplementary Information [file 41467_2017_2167_MOESM1_ESM.pdf]

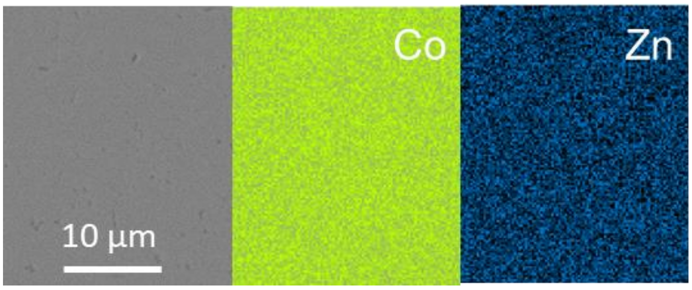

Supplementary Figure 1 | The EDS chemical mappings of cobalt and zinc in a Co_5Zn_{21} ribbon.

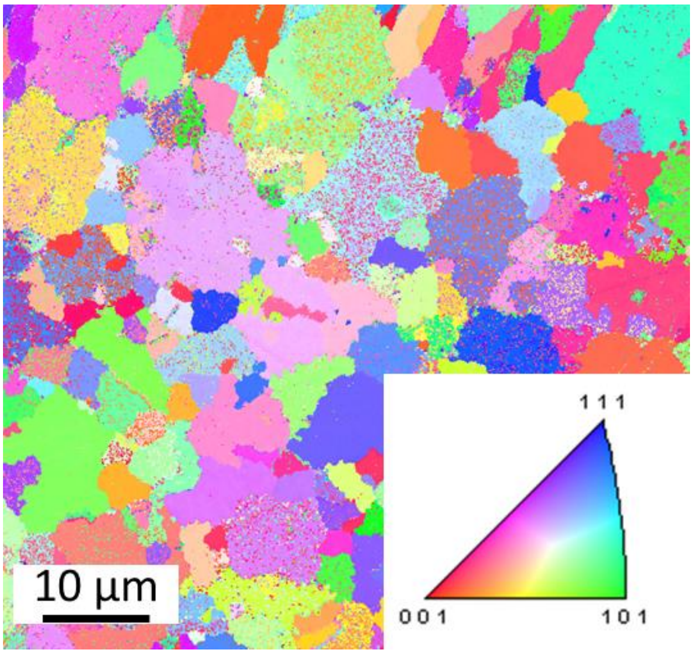

Supplementary Figure 2 | EBSD inverse pole figure mapping of the Co_5Zn_{21} precursor alloy. The average grain size is about 10 µm which is orders of magnitude largers than the nanopore sizes of the dealloyed nanoporous Co.

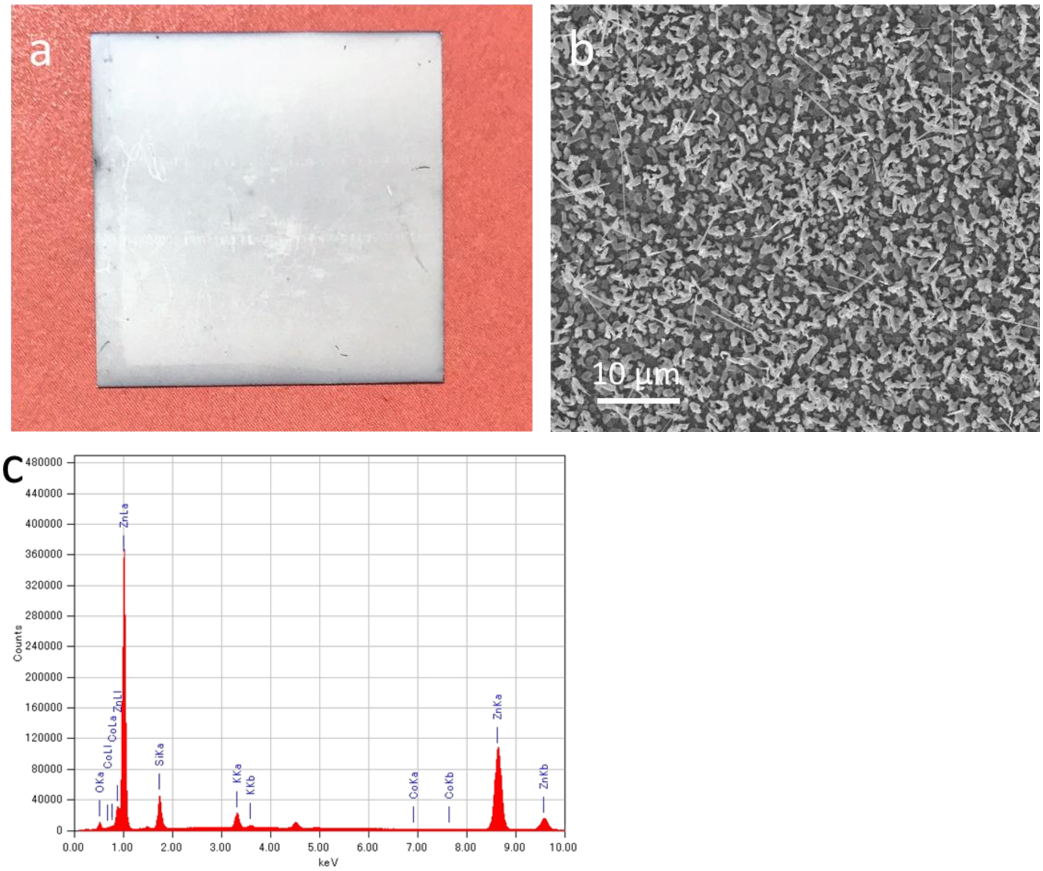

Supplementary Figure 3 | The recovered Zn after dealloying process. (a) The photo of the recovered Zn on the glass substrate. (b) SEM image of the recovered Zn. (c) Energy dispersive X-ray spectrum of the recovered zinc. The signals of Si, O and K originate from the glass substrate.

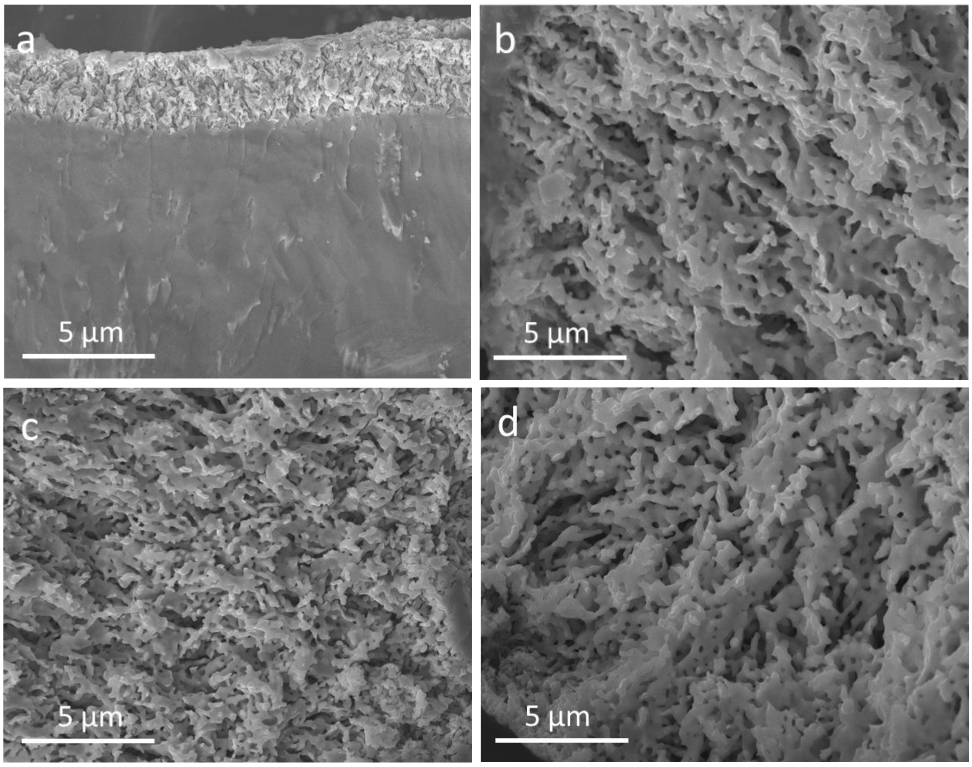

Supplementary Figure 4 | The cross-sectional SEM images of dealloyed nanoporous cobalt. (a)-(d) The cross-section of nanoporous cobalt at 773 K and 100 Pa for various dealloying time of 5 min, 20 min, 40 min, and 120 min, respectively.

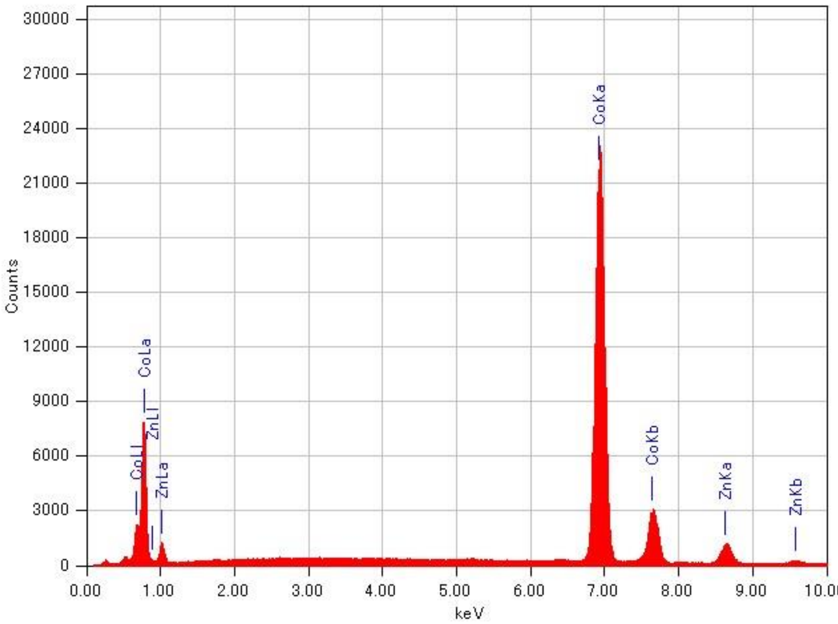

Supplementary Figure 5 | Energy dispersive X-ray spectrum of nanoporous cobalt. The EDS result demonstrates that only about 6 at. % zinc remains in the nanoporous structure after dealloying for 20 min at 773 K and 100 Pa.

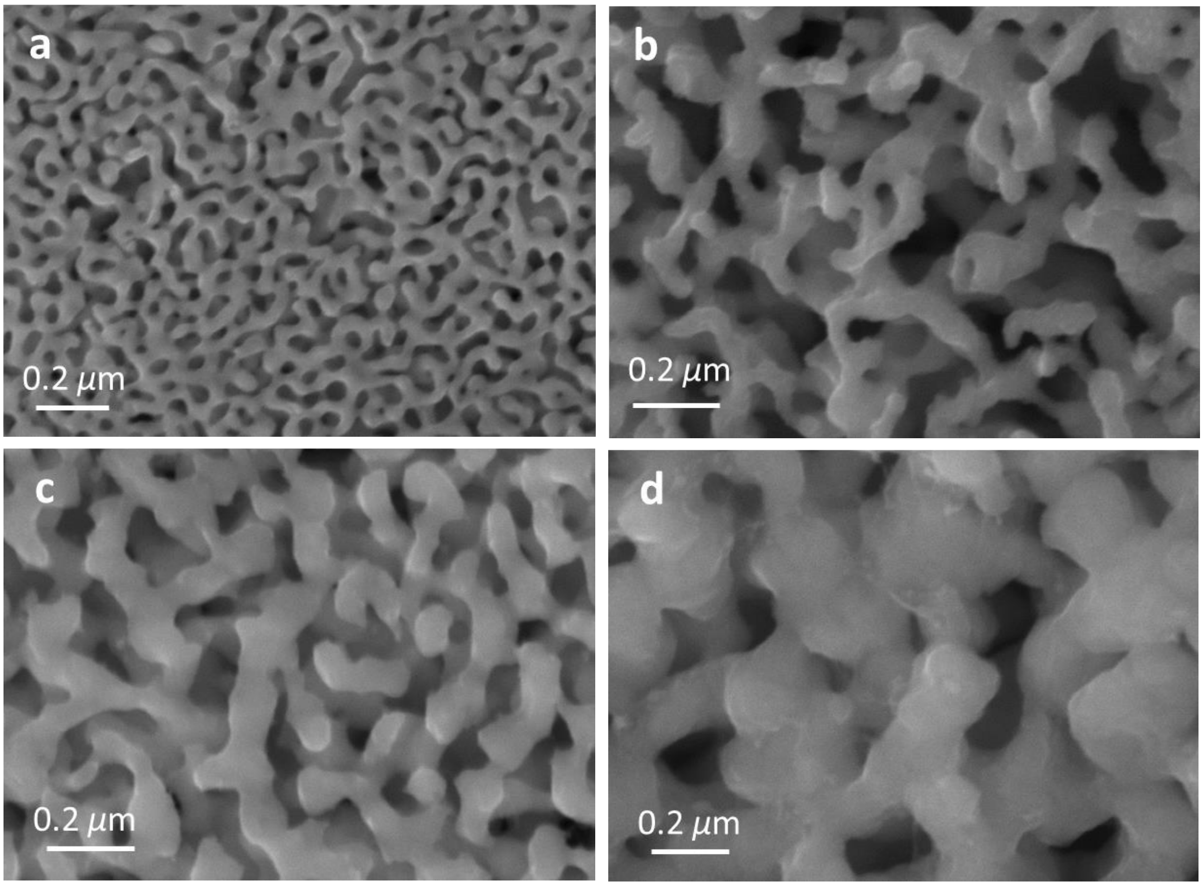

Supplementary Figure 6 | SEM images of nanoporous cobalt with dealloying time of 30 min under a low-vacuum (100 Pa) condition. From (a) to (d), the dealloying temperature is 723 K, 773 K, 873 K, 923 K, respectively.

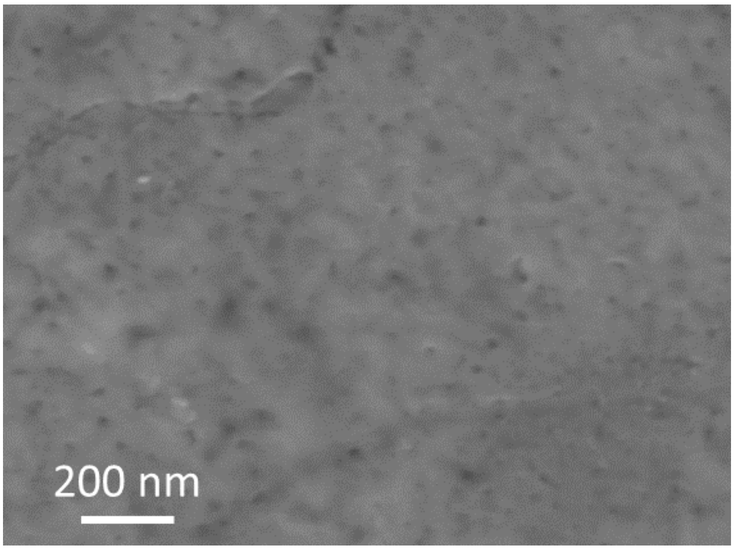

Supplementary Figure 7 | The surface of dealloyed Co_5Zn_{21} alloy. The surface morphology of the Co_5Zn_{21} alloy at 673 K and 100 Pa for 20 min. Well developed nanoporosity cannot be seen at the lower temperature and lower vacuum.

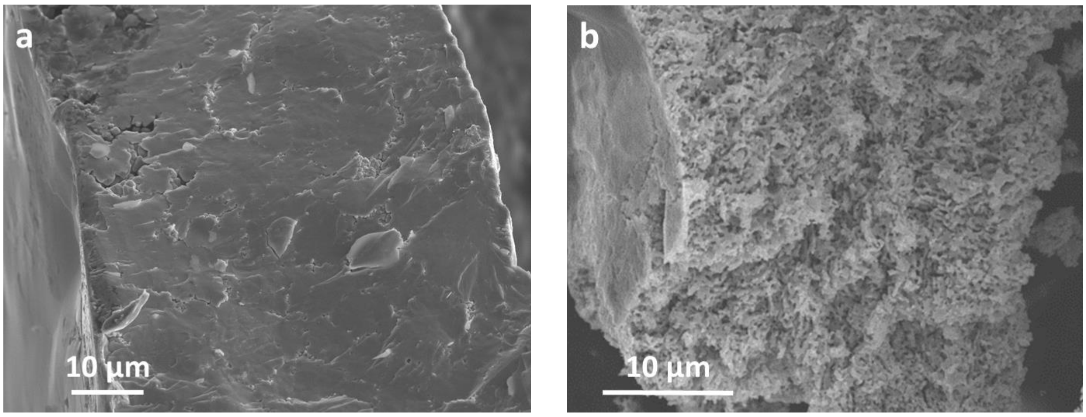

Supplementary Figure 8 | Cross-sectional SEM images of precursor and dealloyed Co_5Zn_{21} samples. (**a**) The cross-section of the precursor Co_5Zn_{21} sample with the thickness of about 60 μm. (**b**) The cross-section of a dealloyed Co_5Zn_{21} sample with the thickness of about 27 μm. The dealloying was conducted at 773 K and 100 Pa for 30 min.

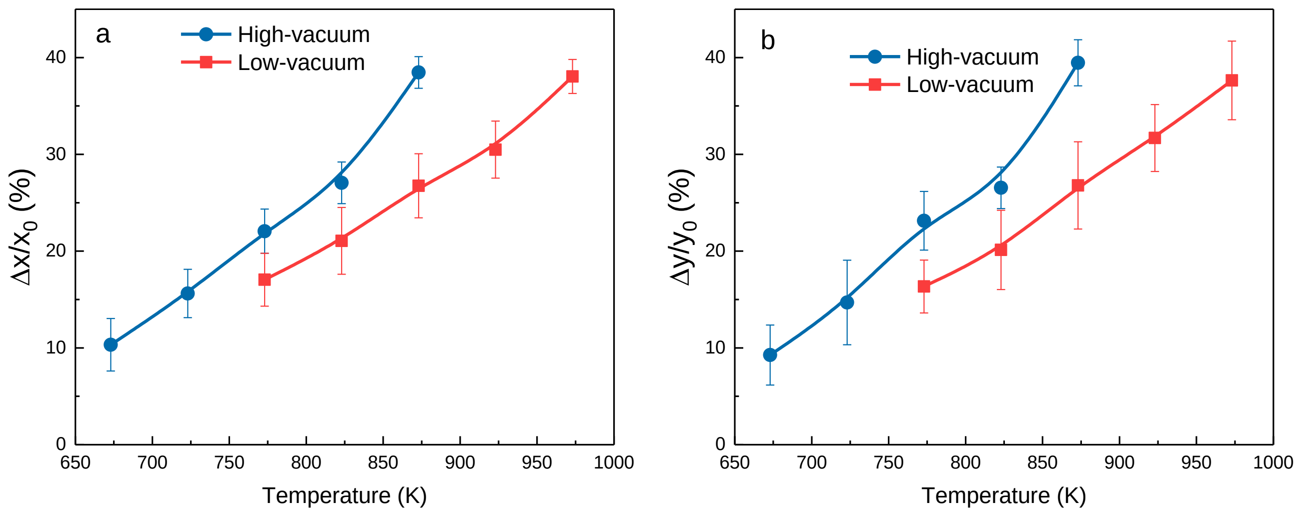

Supplementary Figure 9 | Relation between dealloying temperature and shrinkage in x/y surface plane. (**a**) The shrinkage along x axis for high- and low-vacuum dealloying conditions. (**b**) The shrinkage along y axis for high- and low-vacuum dealloying conditions.

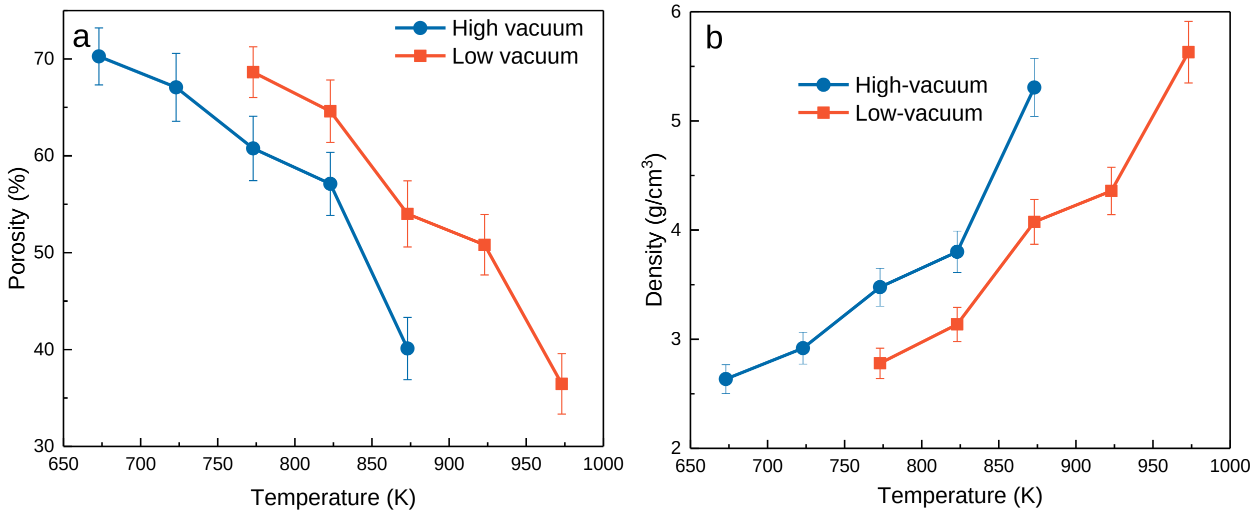

Supplementary Figure 10 | Relation of dealloying temperature with porosity and density. (**a**) The evolution of porosity with temperature in high- and low-vacuum dealloying conditions. (**b**) Correlation between density and temperature in high- and low-vacuum dealloying conditions.

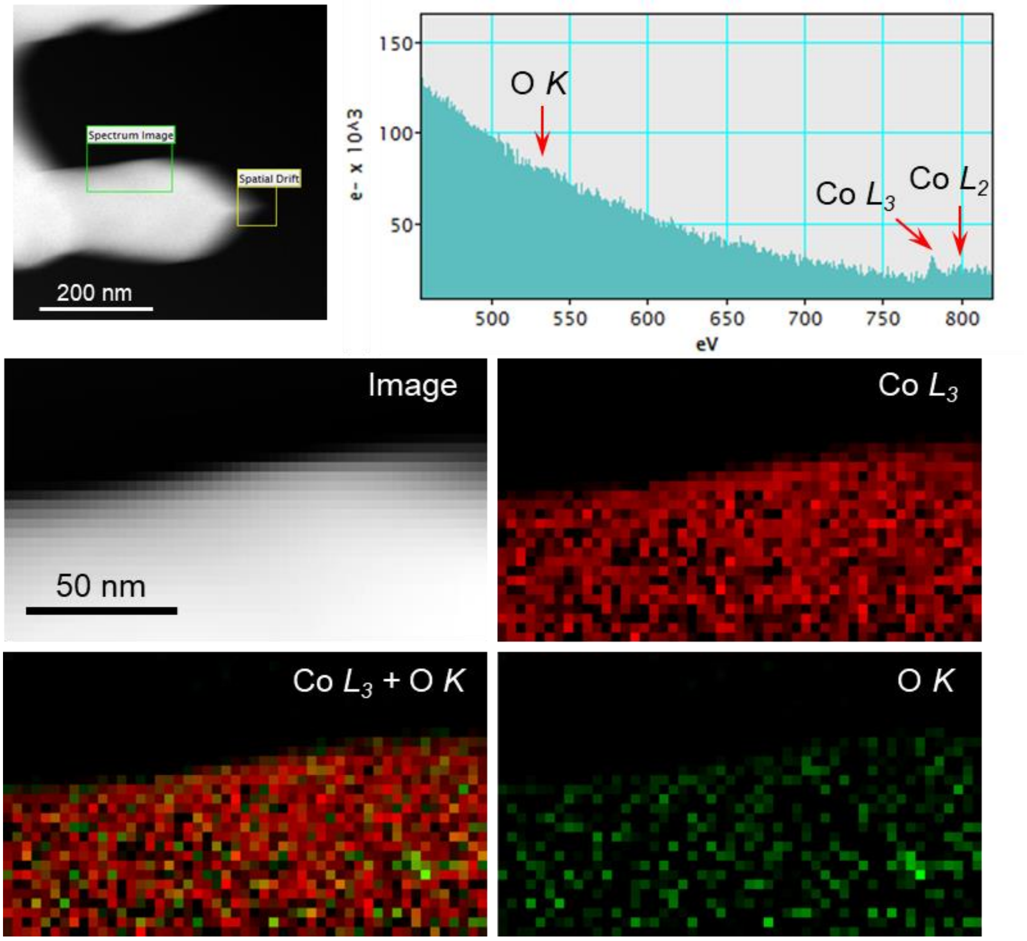

Supplementary Figure 11 | EELS mappings and spectrum of nanoporous cobalt prepared by dealloying at 923 K and 100 Pa for 30 min.

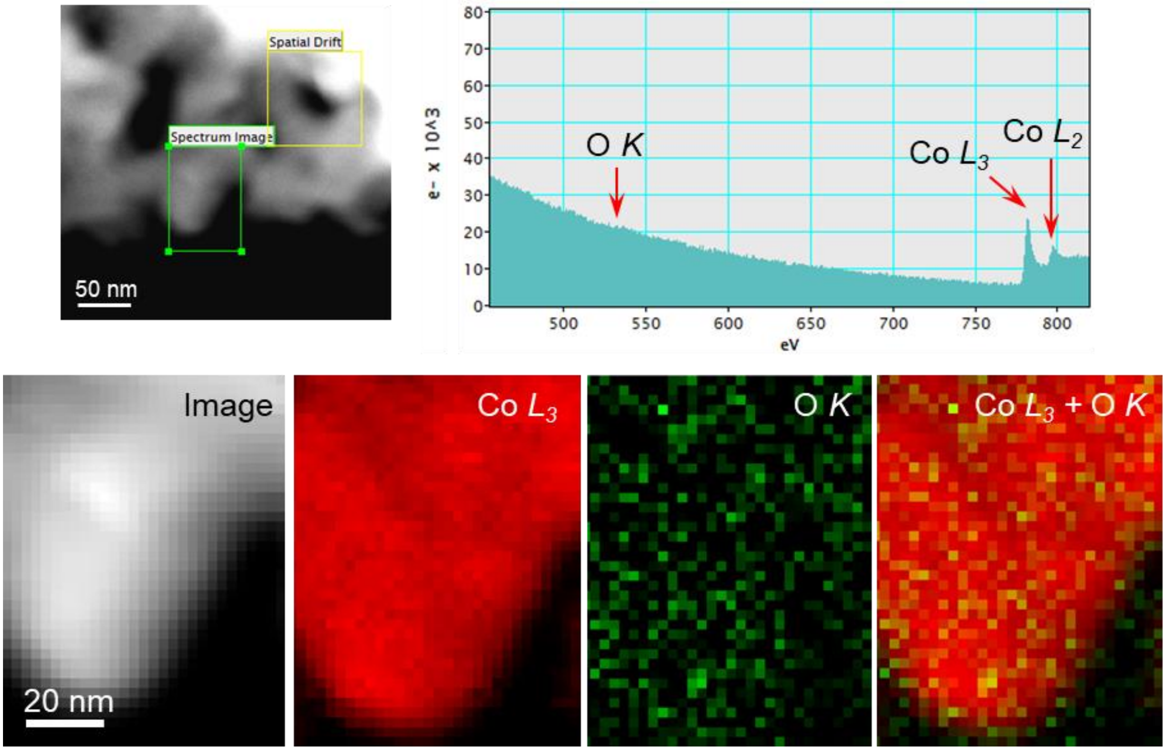

Supplementary Figure 12 | EELS mappings and spectrum of nanoporous cobalt prepared by dealloying at 873 K and 6×10^{-3} Pa for 30 min.

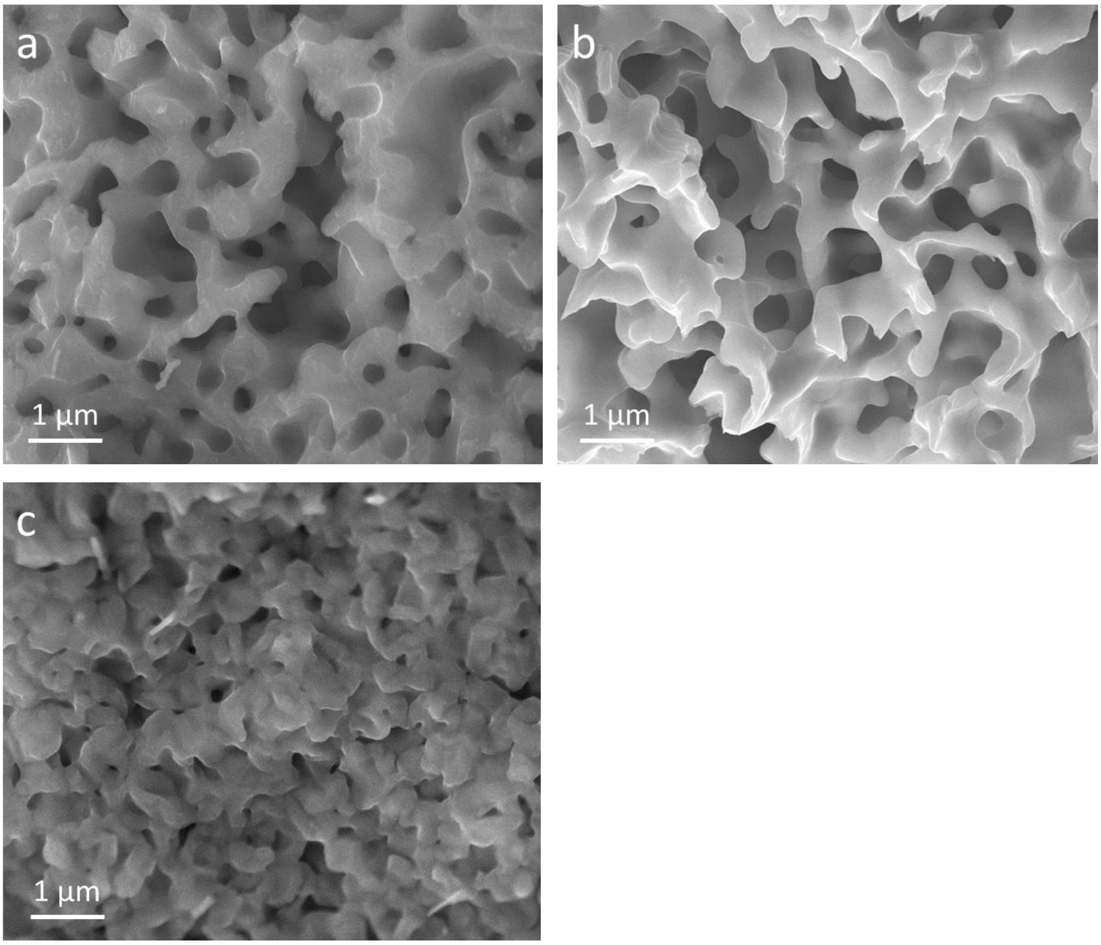

Supplementary Figure 13 | SEM images of nanoporous Ti, Ni and Si prepared by VPD. (a) The morphology of nanopourous Ti with dealloying time of 60 min at 873 K and 100 Pa. (b) The morphology of nanoporous Ni with dealloying time of 60 min at 773 K and 100 Pa. (c) The morphology of nanoporous Si with dealloying time of 30 min at 823 K and 100 Pa.
